# Delay Analysis for End-to-End Synchronous Communication in Monitoring Systems

**DOI:** 10.3390/s18113615

**Published:** 2018-10-24

**Authors:** Junwei Cao, Yuxin Wan, Haochen Hua, Yuchao Qin

**Affiliations:** Research Institute of Information Technology, Tsinghua University, Beijing 100084, China; jcao@tsinghua.edu.cn (J.C.); wanyx04@163.com (Y.W.); qinyc17@mails.tsinghua.edu.cn (Y.Q.)

**Keywords:** smart grid, measurement and control system, performance analysis, end-to-end communication

## Abstract

With the rapid development of smart grid technologies, communication systems are further integrated in the existing power grids. The real-time capability and reliability of the power applications are receiving increasing concerns. Thus, it is important to measure the end-to-end delay in communication systems. The network calculus theory has been widely applied in the communication delay measuring tasks. However, for better operation performance of power systems, most power applications require synchronous data communication, in which the network calculus theory cannot be directly applied. In this paper, we expand the network calculus theory such that it can be used to analyze the communication delay for power applications in smart grids. The problem of communication delay calculation for the synchronization system is converted into a maximum path problem in graph theory. Finally, our theoretical results are compared with the experimental ones obtained with the network simulation software EstiNet. The simulation results verify the feasibility and effectiveness of the proposed method.

## 1. Introduction

With the development of modern communication, computing, network and control technologies, the applications of information technology continue to expand. The combination of information and energy technology has become an inevitable trend of the development for future power systems. This combination has also spawned a new concept: smart grid, which is able to utilize advanced information technology to improve energy management [1,2,3,4]. With smart grid technologies, we are able to control energy flows in power systems more efficiently and precisely.

Communication networks play a key role in the operation and management of smart grids [5,6]. In the monitoring system of a smart grid, a large amount of data needs to be processed and analyzed for the control and dispatch of power systems. The performance of the end-to-end communication of network has an important impact on the real-time capability and reliability of the monitoring system. The end-to-end performance of a communication network concerns both network transmission performance and computational performance. In addition, based on the analysis of smart grid wide-area monitoring cases in [7,8], the data synchronization performance is also one of the basic requirements in smart grid monitoring systems.

The monitoring system for a smart grid is a typical network computing system [9]. For analysis, the network computing system can be regarded as a service system. Stochastic queuing theory, which is developed based on theories of Poisson process and Markov process, plays an important role in the performance analysis of network service systems such as telephone and telegraph networks [10]. However, with the development of computer network systems, network structures and network applications have become more complicated and diverse. The traffic flow in communication networks exhibits properties different from those of Poisson and Markov processes. Therefore, the stochastic queuing theory would produce large deviations when applied to analyze the modern computer networks [11]. On the other hand, the stochastic queuing theory can only provide limited performance metrics, such as the average waiting time and variance of the service system. It cannot be used to obtain the deterministic analysis of the system performance. In order to tackle more complicated analysis of modern network service systems, network calculus theory was proposed [12,13].

The network calculus theory can be applied to the performance analysis for service systems. It can be divided into two branches: deterministic network calculus theory and stochastic network calculus theory; see, e.g., [12,14]. The deterministic network calculus theory can be used to calculate the upper and lower bounds for different kinds of maximum performance of a service system. For example, the maximum delay, the maximum backlog, etc. The stochastic network calculus theory is able to provide the probability distributions for the performance boundaries of a service system, such as the distribution of the maximum delay and the distribution of the maximum backlog. Within the field of smart grids, there are also a number of related research outputs based on communication networks and power grids [15,16,17]. In [18,19], network calculus theory is used to construct a reliability model for a power system which consists of conventional power generation devices, loads and renewable energy sources such as photovoltaic panels and wind power generators, achieving better utilization of renewable energy in smart grids. In [20], network calculus theory is employed to calculate the performance of communication systems in home area networks. Targeting at the operational stability and security of power systems, a bounded model of communication delay is proposed based on network calculus theory in [21].

Despite the great success in applications within smart grids, network calculus theory is not able to handle the communication system with synchronous computing requirements [7]. Unfortunately, there are vast data synchronization requirements in smart grid applications. For a communication system which requires synchronous computing, the end-to-end delay of the system is not only related to the transmission delay, but also related to the difference of delay in different channels, which is not considered in network calculus theory. Thus, deterministic network calculus theory cannot be directly used to provide an estimation for the upper bound of the smart grid monitoring system’s end-to-end delay.

In this paper, based on network calculus theory and the analysis for delay of synchronous communication system, it is shown that the problem to calculate the upper bound for end-to-end delay in the synchronous communication system can be transformed to a general maximum/shortest path selection problem in graph theory. Next, we propose a new method to provide an estimation for the upper bound of the smart grid monitoring system’s end-to-end delay. To show the feasibility and effectiveness of our proposed method, several numerical simulations are performed.

The importance and contributions of this paper are stated as follows:

(1) A class of typical communication model for the monitoring system is investigated in this paper. The transmission network in the smart grid monitoring system is modeled as a transmission service node, such that network calculus theory can be applied. In this sense, the analysis methods proposed in this paper can be used under most scenarios of monitoring systems in the field of smart grid.
(2) It is notable that due to the synchronous property of the smart grid applications, the original network calculus theory cannot be directly applied in the delay analysis discussed in this paper. Based on the network calculus theory, an upper bound for the end-to-end delay in the synchronous communication system is derived. The simulations demonstrate the feasibility of the proposed method. With the development of the smart grid systems, there will be more applications based on the monitoring systems, and the theoretical results obtained in this paper can be utilized to improve the reliability and efficiency of the smart grid systems.
(3) In this paper, three theorems are proposed as our main results. In Theorem 1, the upper bound for the transmission delay in a transmission service node with strict service curve is derived. The data transmission delay in different time periods are discussed in detail. In Theorem 2, the formula for upper bound of system’s delay with multiple times of data exchange is derived. In Theorem 3, a general upper bound for transmission delay in the considered system is proposed.

The rest of the paper is organized as follows: Section 2 introduces the considered synchronous calculation model. Section 3 presents the theoretical analysis for the upper bound of the end-to-end communication delay in the considered model. Section 4 provides several numerical simulations to evaluate our main results. Finally, a conclusion is given in Section 5.

## 2. Typical Models for the Synchronous Communication Systems

**Figure 1:**
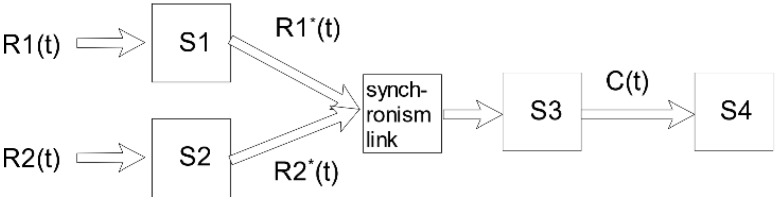
Synchronous calculation and transmission service model for the monitoring system.

Consider the synchronous calculation and transmission model in Figure 1. Such a model has been extensively studied in [7], and it is a typical synchronous communication model for monitoring systems in smart grids.

In Figure 1, $R_{1}\left(t\right)$ and $R_{2}\left(t\right)$ stand for the input of two sets of monitoring data, and the corresponding arrival curves of the monitoring data are $\alpha_{1}$ and $\alpha_{2}$. Similar to the data arrival curves proposed in network calculus theory [22], $S_{1}$ and $S_{2}$ represent for the equivalent transmission service nodes where the monitoring data go through the control center, and the corresponding service curves are denoted as $\beta_{1}$ and $\beta_{2}$. Here, the data arrival curve is the characteristic curve which is used to describe the monitoring data. We denote $S_{3}$ as an equivalent computing service node, with the scaling function being S(n) and the calculation service curve being C. $S_{4}$ is denoted as a follow-up service model, with its the service curve denoted as $\beta_{4}$. Before $R_{1}\left(t\right)$ and $R_{2}\left(t\right)$ enter into $S_{3}$, they go through a synchronous link which causes the equivalent transmission service curve changing. Let us denote $\beta_{1}^{\prime }$ and $\beta_{2}^{\prime }$ as the equivalent transmission service curves after the synchronization link. Here, $\beta_{1}^{\prime }$ and $\beta_{2}^{\prime }$ are functions of $\beta_{1}$ and $\beta_{2}$, i.e., $\beta_{1}^{\prime }=F_{1}\left(\beta_{1},\beta_{2}\right),$ and $\beta_{2}^{\prime }=F_{2}\left(\beta_{1},\beta_{2}\right).$

The smart grid’s wide area measurement system has three components: power monitor unit (PMU), communication network and controller. The operation parameters of the utility grid within different regions are measured by PMU. Based on the time scale from the global positioning system, such data is sent to the control center for analysis and procession. Let $G_{1}\left(t\right)$ and $G_{2}\left(t\right)$ be defined as the amount of data that is generated by Sensor 1 and Sensor 2 with time scale t, respectively. Then, we have:

$$R_{1}\left(t\right)=\int_{0}^{t}G_{1}\left(t\right)dt,$$

and:

$$R_{2}\left(t\right)=\int_{0}^{t}G_{2}\left(t\right)dt.$$

Next, the flow ratio is defined. Here, we assume that the sensor has a synchronous clock, and the time scale of data is marked at the same time, i.e., if $G_{2}\left(t\right)\neq 0$, then $G_{1}\left(t\right)\neq 0$. We assume that $G_{2}\left(t\right)\neq 0$, and we define $\rho_{max}≜max\frac{G_{1}\left(t\right)}{G_{2}\left(t\right)},$ and $\rho_{min}≜min\frac{G_{1}\left(t\right)}{G_{2}\left(t\right)}.$ The data arriving at $S_{3}$ is synchronized. The aggregate received data calculation service curve is C. Then, we have the minimal computational service curve for $R_{1}$, which is $\frac{\rho_{min}}{1+\rho_{min}}C.$ Similarly, for $R_{2}$, the minimal computational service curve is $\frac{1}{1+\rho_{max}}C.$

According to the above assumptions and calculation model of unified service transmission, the equivalent end-to-end service model for monitoring data $R_{1}$ is obtained as follows:

(1) $$\beta_{1}^{\prime }⊗\frac{\rho_{min}}{1+\rho_{min}}\left[C⊗\underline{S^{-1}}\left(\beta_{4}\right)\right]=F_{1}\left(\beta_{1},\beta_{2}\right)⊗\frac{\rho_{min}}{1+\rho_{min}}\left[C⊗\underline{S^{-1}}\left(\beta_{4}\right)\right],$$

where the notation ⊗ stands for the convolutional operator. Similarly, the equivalent end-to-end service model for monitoring data $R_{2}$ can be obtained as follows:

$$\beta_{2}^{\prime }⊗\frac{1}{1+\rho_{max}}\left[C⊗\underline{S^{-1}}\left(\beta_{4}\right)\right]=F_{2}\left(\beta_{1},\beta_{2}\right)⊗\frac{1}{1+\rho_{max}}\left[C⊗\underline{S^{-1}}\left(\beta_{4}\right)\right].$$

In real-world scenarios, the monitoring data is normally the same. Hence, $\rho_{max}=\rho_{min}=1.$ In the above model, both sensors $R_{1}$ and $R_{2}$ reach the service node $S_{3}$ simultaneously due to the synchronization process. Thus, their processing time is also the same. After the calculation processing, the data of sensor $R_{1}$ and $R_{2}$ go through the same service node $S_{4}$ with the same time delay. This refers to the so-called synchronization property; see, [7], and the references therein.

Based on the synchronous property, the end-to-end delay of the synchronization system can be analyzed. We assume that the data of sensor $R_{1}$ always arrives earlier than that of $R_{2}$ during a given time period [0, t]. This means that the data of $R_{1}$ always waits for that of $R_{2}$ in the section of synchronization. As soon as the data of $R_{2}$ arrives, it can be input into the service node $S_{3}$. Hence, regarding $R_{2}$ the equivalent service curve is not changed via the synchronized transmission link, and we have $\beta_{2}^{\prime }=F_{2}\left(\beta_{1},\beta_{2}\right)=\beta_{2}.$ According to [7], the end-to-end service curve of $R_{2}$ can be expressed as:

(2) $$\beta_{2}⊗\frac{1}{1+\rho_{max}}\left[C⊗\underline{S^{-1}}\left(\beta_{4}\right)\right].$$

According to the service theorems in network calculus theory [22] and the obtained public network flow model, $\beta_{2}$ and $\beta_{4}$ can be obtained directly. We have the upper bound of the end-to-end delay of $R_{2}$ being:

(3) $$h\left(\alpha_{2},\beta_{2}⊗\frac{1}{1+\rho_{max}}\left[C⊗\underline{S^{-1}}\left(\beta_{4}\right)\right]\right).$$

However, the equivalent transmission service curve of $R_{1}$ has been changed, since the data of $R_{1}$ cannot go through service node $S_{3}$ until the arrival of the $R_{2}$. Then, we have $\beta_{1}^{\prime }=F_{1}\left(\beta_{1},\beta_{2}\right)\neq \beta_{1}.$ In fact, due to the waiting time of data, the delay of $R_{1}$’s data may increase. Then, we have $\beta_{1}^{\prime }\leq \beta_{1}.$ According to the synchronization property, the end-to-end delay of $R_{1}$ and $R_{2}$ are the same, even if $\beta_{1}^{\prime }$ is unable to be obtained. The upper bound of the end-to-end delay of $R_{1}$ can be expressed by (3).

The problem is that the conclusion of (3) was tenable, only if we assume that $R_{1}$’s data always arrives earlier than $R_{2}$’s. If $R_{1}$’s data arrives later than $R_{2}$’s after time $t^{*}$, the expression of the end-to-end service curve of $R_{1}$ is presented as (4), which shall be investigated in Section 3:

(4) $$\beta_{1}⊗\frac{\rho_{min}}{1+\rho_{min}}\left[C⊗\underline{S^{-1}}\left(\beta_{4}\right)\right].$$

Although the data of $R_{1}$ arrives later, most of the equivalent transmission curve of $R_{1}$ has already changed before $t^{*}$. So, the service curve of $R_{1}$ cannot be $\beta_{1}$, i.e., (4) cannot be the equivalent transmission service curve to $R_{1}$. In order to calculate the end-to-end delay in this case, the delay theory of the suspension service system is discussed in Section 3.

## 3. Calculation of Equivalent Delay of Monitoring System

The main results of the equivalent delay calculation are provided as three theorems in this section.

### 3.1. Delay Theorem of Suspension Service System

**Theorem** **1.** *Consider an input* R(t) *through a service node which has the strict service curve β(t). The system does not provide any service during $t_{1}<t<t_{2}$. We assume that $R^{*}(t_{1})$ is known and time delay of the original system is d(t). For $t>t_{1}$, the delay is denoted as $d^{\prime }\left(t\right)$. Then, $d^{\prime }\left(t\right)$ satisfies the following inequality:*

(5) $$d^{\prime }\left(t\right)\leq d\left(t\right)\vee \left[inf\{\tau \geq 0,\;R\left(t\right)-R^{*}(t_{1})\leq \beta \left(\tau \right)\}+t_{2}-t\right],$$

*where ‘∨’ refers taking the maximum value.*

The proof of Theorem 1 is given in Appendix A; see, Appendix A.1.

Before time $t^{*}$, data of $R_{1}$ always arrives earlier than that of $R_{2}$. But after time $t^{*}$, on the contrary, $R_{2}$’s data arrives earlier. The service received by $R_{1}$ can be equivalent to a suspended service system, and the suspended time period is $d_{1}\left(t^{*}\right)<t<d_{2}\left(t^{*}\right)$, where $d_{1}\left(t^{*}\right)$ and $d_{2}\left(t^{*}\right)$ are obtained from (2) and (4), respectively. Because of the synchronization, the data which was supposed to be processed in $d_{1}\left(t^{*}\right)$ by $R_{1}$ will not be completed until $d_{2}\left(t^{*}\right)$, and this is equivalent to the system being suspended for $R_{1}$. 

### 3.2. Synchronization System Delay Analysis

Since the data sent by sensors is in accordance with a fixed sampling interval, we denote the time scale of such data as $T_{1}<T_{2}<T_{3}<\cdots <T_{n}$. Then, we have:

$$R_{1}\left(t\right)=\overset{m(T_{m}\leq t<T_{m+1})}{\underset{k=1}{\sum }}G_{1}\left(T_{k}\right),\;\mathrm{and}\;R_{2}\left(t\right)=\overset{m(T_{m}\leq t<T_{m+1})}{\underset{k=1}{\sum }}G_{2}\left(T_{k}\right).$$

Let us introduce the following definitions.

**Definition** **1.** *Let us denote $\beta_{R1}^{1}$, $\beta_{R1}^{2}$, $\beta_{R2}^{1}$, $\beta_{R2}^{2}$ as service curves of flow $R_{1}$ and flow $R_{2}$ before and after the synchronous link, respectively, then we have $\beta_{R1}^{1}=\beta_{1},\;\beta_{R2}^{1}=\beta_{2},$ and:*

$$\beta_{R1}^{2}=\frac{\rho_{min}}{1+\rho_{min}}\left[C⊗\underline{S^{-1}}\left(\beta_{4}\right)\right],\;\beta_{R2}^{2}=\frac{1}{1+\rho_{max}}\left[C⊗\underline{S^{-1}}\left(\beta_{4}\right)\right].$$

**Definition** **2.** *Denote $d_{i}^{x}\left(t\right)$ as the delay upper bound calculated by the equivalent service model using flow i, after the data arrival sequence changes for the x-th time. Denote $D_{i}\left(t\right)$ as the upper bound of the system delay before time t, when the data arrival order has already changed i times. Then, we have:*

$$D_{0}\left(t\right)=d_{1}^{0}\left(t\right)\vee d_{2}^{0}\left(t\right),$$

$$D_{1}\left(t\right)=d_{1}^{1}\left(t\right)\vee d_{2}^{1}\left(t\right),$$

⋯

$$D_{n}\left(t\right)=d_{1}^{n}\left(t\right)\vee d_{2}^{n}\left(t\right).$$

We assume that the flow arrival curves for $R_{1}$ and $R_{2}$ are $\alpha_{1}$ and $\alpha_{2}$, respectively, and data of $R_{1}$ always arrives earlier than that of $R_{2}$ at the synchronization service node no later than time $T_{x_{1}}$. The flow delay of $R_{2}$ is $d_{2}^{0}\left(t\right)$. If there is no synchronization mechanism, flow delay of $R_{1}$ is $d_{1}^{0}\left(t\right)$. Since $R_{1}$’s data always arrives earlier than $R_{2}$’s, the system delay should be $R_{2}$’s delay which is $d_{2}^{0}\left(t\right)$. According to network calculus theory, we have $d_{2}^{0}\left(t\right)=h\left(\alpha_{2},\beta_{R2}\right).$

After time $T_{x_{1}}$, $R_{2}$’s data arrives earlier than $R_{1}$’s at the synchronization service node. Thereafter, the delay of $R_{1}$ should be taken as the system delay. However, due to the waiting time of flow $R_{1}$, the original system delay $\beta_{R1}$ has changed. According to the analysis in Section 3.1, the equivalent time period of flow $R_{1}$ is obtained as follows:

(6) $$T_{x_{1}}+d_{1}^{0}\left(T_{x_{1}}\right)<t<T_{x_{1}}+d_{2}^{0}\left(T_{x_{1}}\right).$$

Flow $R_{1}$ can be seen as the output of the suspensive service $\beta_{R1}^{2}$ which comes from service $\beta_{R1}^{1}$ first. The suspension time for service $\beta_{R1}^{2}$ is the length of the time period given in (6). Assume that the data from flow $R_{1}$ passes by $\beta_{R1}^{1}$, the output is $R_{1}^{1}\left(t\right)$ at time t, and the data from flow $R_{1}$ reaches the synchronization link at time t’. Obviously, $R_{1}^{1}\left(t^{\prime }\right)=R_{1}\left(t\right).$

Since the considered system is a periodic sampling monitoring system, data can only be transmitted in a fixed time period. The delay of the data transmission needs to be taken into consideration only when data is transmitted. Let us define $t=T_{m}.$. Thus, $t^{\prime }$ defined above stands for the time when data with time scale $T_{m}$ reaches the synchronize link.

Assuming that the output of flow $R_{1}$ by service $\beta_{R1}^{1}$, synchronization and service $\beta_{R1}^{2}$ at time $T_{m}$ is $R_{1}^{*}(t)$, then, for the suspended starting point $T_{x_{1}}+d_{1}^{0}\left(T_{x_{1}}\right)$, we have:

$$R_{1}^{*}\left(T_{x_{1}}+d_{1}^{0}\left(T_{x_{1}}\right)\right)=\overset{x_{1}}{\underset{k=1}{\sum }}G_{1}\left(T_{k}\right).$$

Discussions for the value of m in $T_{m}$ is given in Appendix A; see, Appendix A.2.

Let us assume that after time scale $T_{x_{2}}$, data $R_{1}$ arrives the synchronization node before data $R_{2}$ with the same time scale. $R_{2}$’s traffic can be seen as the output of the suspensive service $\beta_{R2}^{1}$ which gets through service $\beta_{R2}^{2}$ first. The suspension time is:

$$T_{x_{2}}+d_{2}^{0}\left(T_{x_{2}}\right)<t<T_{x_{2}}+d_{1}^{1}\left(T_{x_{2}}\right).$$

Therefore, $T_{m}>T_{x_{2}}$, the upper bound of data delay be expressed as:

$$d_{2}^{2}\left(T_{m}\right)=d_{2}^{0}\left(T_{m}\right)\vee d_{1}^{1}\left(T_{m}\right)\vee \left[\varphi \left(m,T_{x_{2}},2\right)+d_{1}^{1}\left(T_{x_{2}}\right)+T_{x_{2}}-T_{m}\right].$$

In accordance with the discussion of above, when $T_{\omega }\geq T_{x_{1}}$:

$$d_{1}^{1}\left(T_{\omega }\right)=d_{1}^{0}\left(T_{\omega }\right)\vee d_{2}^{0}\left(T_{\omega }\right)\vee \left[\varphi \left(\omega ,T_{x_{1}},1\right)+d_{2}^{0}\left(T_{x_{1}}\right)+T_{x_{1}}-T_{\omega }\right].$$

If $d_{1}^{1}\left(T_{x_{2}}\right)=d_{2}^{0}\left(T_{x_{2}}\right)$, then the equivalent model of the service is not suspended, so $\left(T_{m}\right)=d_{2}^{0}\left(T_{m}\right).$ Otherwise, we can obtain:

$$\begin{array}{cc} d_{2}^{2}\left(T_{m}\right)=d_{2}^{0}\left(T_{m}\right)\vee d_{1}^{0}\left(T_{m}\right) & \vee \left[\varphi \left(m,T_{x_{1}},1\right)+d_{2}^{0}\left(T_{x_{1}}\right)+T_{x_{1}}-T_{m}\right]\\ & \vee \left[\varphi \left(m,T_{x_{2}},2\right)+d_{1}^{0}\left(T_{x_{2}}\right)+T_{x_{2}}-T_{m}\right]\\ & \vee \left[\varphi \left(m,T_{x_{2}},2\right)+\varphi \left(T_{x_{2}},T_{x_{1}},1\right)+d_{2}^{0}\left(T_{x_{1}}\right)+T_{x_{1}}-T_{m}\right]. \end{array}$$

The problem is that the time of $x_{1}$ and $x_{2}$ cannot be obtained with the existing theory in network calculus [22]. The maximum of:

$$d_{1}^{1}\left(T_{\omega }\right)=d_{1}^{0}\left(T_{\omega }\right)\vee d_{2}^{0}\left(T_{\omega }\right)\vee \left[\varphi \left(\omega ,T_{x_{1}},1\right)+d_{2}^{0}\left(T_{x_{1}}\right)+T_{x_{1}}-T_{\omega }\right].$$

is the bound of the system, which is:

$${Max}_{1\leq x_{1},x_{1}+2\leq \omega }\left[\varphi \left(\omega ,T_{x_{1}},1\right)+d_{2}^{0}\left(T_{x_{1}}\right)+T_{x_{1}}-T_{\omega }\right].$$

So, for $T_{x_{2}}\geq T_{\omega }\geq T_{x_{1}}$,

$$\begin{array}{cc} d_{1}^{1}\left(T_{\omega }\right) & \leq d_{1}^{0}\left(T_{\omega }\right)\vee d_{2}^{0}\left(T_{\omega }\right)\vee {Max}_{1\leq \theta ,\theta +2\;\leq \omega }\left[\varphi \left(\omega ,T_{\theta },1\right)+d_{2}^{0}\left(T_{\theta }\right)+T_{\theta }-T_{\omega }\right]\\ & \leq d_{1}^{0}\left(T_{\omega }\right)\vee d_{2}^{0}\left(T_{\omega }\right)\vee {Max}_{1\leq \theta \;\leq \omega }\left[\varphi \left(\omega ,T_{\theta },1\right)+d_{2}^{0}\left(T_{\theta }\right)+T_{\theta }-T_{\omega }\right]. \end{array}$$

Furthermore, for $T_{m}>T_{x_{2}}$:

$$\begin{array}{cc} d_{2}^{2}\left(T_{m}\right)\leq d_{2}^{0}\left(T_{m}\right) & \vee d_{1}^{0}\left(T_{m}\right)\\ & \vee {Max}_{1\leq \theta_{1}\leq m}[\varphi \left(m,T_{\theta_{1}},1\right)+d_{2}^{0}\left(T_{\theta_{1}}\right)+T_{\theta_{1}}-T_{m}]\\ & \vee {Max}_{1\leq \theta_{2}\leq m}\left[\varphi \left(m,2\right)+d_{1}^{0}\left(T_{\theta_{2}}\right)+T_{\theta_{2}}-T_{m}\right]\\ & \vee {Max}_{1\leq \theta_{1}\leq \theta_{2}\leq m}\left[\varphi \left(m,T_{\theta_{2}},2\right)+\varphi \left(T_{\theta_{2}},T_{\theta_{1}},1\right)+d_{2}^{0}\left(T_{\theta_{1}}\right)+T_{\theta_{1}}-T_{m}\right]. \end{array}$$

Given $\theta_{1}=\theta_{2}$, according to (A1), we have:

$$\begin{array}{c} {Max}_{1\leq \theta_{1}=\theta_{2}\leq m}\left[\varphi \left(m,T_{\theta_{2}},2\right)+\varphi \left(T_{\theta_{2}},T_{\theta_{1}},1\right)+d_{2}^{0}\left(T_{\theta_{1}}\right)+T_{\theta_{1}}-T_{m}\right]=\\ {Max}_{1\leq \theta_{1}\leq m}\left[\varphi \left(m,T_{\theta_{1}},1\right)+d_{2}^{0}\left(T_{\theta_{1}}\right)+T_{\theta_{1}}-T_{m}\right]. \end{array}$$

So, for $T_{m}\geq T_{x_{2}},$ we have:

$$\begin{array}{cc} d_{2}^{2}\left(T_{m}\right)\leq d_{2}^{0}\left(T_{m}\right)\vee d_{1}^{0}\left(T_{m}\right) & \vee {Max}_{1\leq \theta_{2}\leq m}[\varphi \left(m,T_{\theta_{2}},2\right)+d_{1}^{0}\left(T_{\theta_{2}}\right)+T_{\theta_{2}}-T_{m}]\\ & \vee {Max}_{1\leq \theta_{1}\leq \theta_{2}\leq m}\left[\varphi \left(m,T_{\theta_{2}},2\right)+\varphi \left(T_{\theta_{2}},T_{\theta_{1}},1\right)+d_{2}^{0}\left(T_{\theta_{1}}\right)+T_{\theta_{1}}-T_{m}\right]. \end{array}$$

For $T_{m}>T_{x_{2}}$, the upper bound of the system delay is $d_{1}^{2}\left(T_{m}\right)$. For $T_{x_{2}}\geq T_{\omega }\geq T_{x_{1}}$, the upper bound of the system delay is $d_{2}^{1}\left(T_{\omega }\right)$. For $T_{x_{1}}\geq T_{\varphi }$, the upper bound of the system delay is $d_{1}^{0}\left(T_{\varphi }\right)$. Then, we have:

$$\begin{array}{cc} d_{1}^{2}\left(T_{m}\right)\leq & {Max}_{1\leq \theta_{2}\leq m}\left[\varphi \left(m,T_{\theta_{2}},1\right)+d_{2}^{0}\left(T_{\theta_{2}}\right)+T_{\theta_{2}}-T_{m}\right]\vee \\ & {Max}_{1\leq \theta_{1}\leq \theta_{2}\leq m}\left[\varphi \left(m,T_{\theta_{2}},1\right)+\varphi \left(T_{\theta_{2}},T_{\theta_{1}},2\right)+d_{1}^{0}\left(T_{\theta_{1}}\right)+T_{\theta_{1}}-T_{m}\right]\vee \\ & d_{2}^{0}\left(T_{m}\right)\vee d_{1}^{0}\left(T_{m}\right), \end{array}$$

And:

$$d_{2}^{1}\left(T_{\omega }\right)\leq d_{1}^{0}\left(T_{\omega }\right)\vee d_{2}^{0}\left(T_{\omega }\right)\vee {Max}_{1\leq \theta \;\leq \omega }\left[\varphi \left(\omega ,T_{\theta },2\right)+d_{1}^{0}\left(T_{\theta }\right)+T_{\theta }-T_{\omega }\right].$$

Therefore, we have

$$\begin{array}{c} D_{1}\left(T_{\omega }\right)=d_{1}^{1}\left(T_{\omega }\right)\vee d_{2}^{1}\left(T_{\omega }\right)\leq {Max}_{1\leq \theta \leq \omega }\left[\varphi \left(\omega ,\theta ,1\right)+d_{2}^{0}\left(T_{\theta }\right)+T_{\theta }-T_{\omega }\right]\vee \\ {Max}_{1\leq \theta \leq \omega }\left[\varphi \left(\omega ,\theta ,2\right)+d_{1}^{0}\left(T_{\theta }\right)+T_{\theta }-T_{\omega }\right]\vee d_{1}^{0}\left(T_{\omega }\right)\vee d_{2}^{0}\left(T_{\omega }\right), \end{array}$$

and:

$$\begin{array}{cc} D_{2}\left(T_{m}\right)= & d_{1}^{2}\left(T_{m}\right)\vee d_{2}^{2}\left(T_{m}\right)\leq d_{2}^{0}\left(T_{m}\right)\vee d_{1}^{0}\left(T_{m}\right)\vee \\ & {Max}_{1\leq \theta_{2}\leq m}\left[\varphi \left(m,T_{\theta_{2}},2\right)+d_{1}^{0}\left(T_{\theta_{2}}\right)+T_{\theta_{2}}-T_{m}\right]\vee \\ & {Max}_{1\leq \theta_{1}\leq \theta_{2}\leq m}\left[\varphi \left(m,T_{\theta_{2}},2\right)+\varphi \left(T_{\theta_{2}},T_{\theta_{1}},1\right)+d_{2}^{0}\left(T_{\theta_{1}}\right)+T_{\theta_{1}}-T_{m}\right]\vee \\ & {Max}_{1\leq \theta_{2}\leq m}\left[\varphi \left(m,T_{\theta_{2}},1\right)+d_{2}^{0}\left(T_{\theta_{2}}\right)+T_{\theta_{2}}-T_{m}\right]\vee \\ & {Max}_{1\leq \theta_{1}\leq \theta_{2}\leq m}\left[\varphi \left(m,T_{\theta_{2}},1\right)+\varphi \left(T_{\theta_{2}},T_{\theta_{1}},2\right)+d_{1}^{0}\left(T_{\theta_{1}}\right)+T_{\theta_{1}}-T_{m}\right]. \end{array}$$

If $\theta_{1}=\theta_{2}$, according to formula (A1), we have:

$$\begin{array}{c} {Max}_{1\leq \theta_{1}=\theta_{2}\leq m}[\varphi \left(m,T_{\theta_{2}},1\right)+\varphi \left(T_{\theta_{2}},T_{\theta_{1}},2\right)+d_{1}^{0}\left(T_{\theta_{1}}\right)+T_{\theta_{1}}-t]=\\ {Max}_{1\leq \theta_{2}\leq m}\left[\varphi \left(m,T_{\theta_{2}},2\right)+d_{1}^{0}\left(T_{\theta_{2}}\right)+T_{\theta_{2}}-t\right], \end{array}$$

$$\begin{array}{c} {Max}_{1\leq \theta_{1}=\theta_{2}\leq m}[\varphi \left(m,T_{\theta_{2}},2\right)+\varphi \left(T_{\theta_{2}},T_{\theta_{1}},1\right)+d_{2}^{0}\left(T_{\theta_{1}}\right)+T_{\theta_{1}}-t]=\\ {Max}_{1\leq \theta_{2}\leq m}\left[\varphi \left(m,T_{\theta_{2}},1\right)+d_{2}^{0}\left(T_{\theta_{2}}\right)+T_{\theta_{2}}-t\right]. \end{array}$$

So:

$$\begin{array}{cc} D_{2}\left(T_{m}\right)\leq & {Max}_{1\leq \theta_{1}\leq \theta_{2}\leq m}\left[\varphi \left(m,T_{\theta_{2}},2\right)+\varphi \left(T_{\theta_{2}},T_{\theta_{1}},1\right)+d_{2}^{0}\left(T_{\theta_{1}}\right)+T_{\theta_{1}}-T_{m}\right]\vee \\ & {Max}_{1\leq \theta_{1}\leq \theta_{2}\leq m}\left[\varphi \left(m,T_{\theta_{2}},1\right)+\varphi \left(T_{\theta_{2}},T_{\theta_{1}},2\right)+d_{1}^{0}\left(T_{\theta_{1}}\right)+T_{\theta_{1}}-T_{m}\right]\vee \\ & d_{2}^{0}\left(T_{m}\right)\vee d_{1}^{0}\left(T_{m}\right). \end{array}$$

### 3.3. Synchronization System Delay Upper Bound Theorem

**Theorem** **2.** *The system’s delay upper bound of the n-th exchange of data arrival sequence before time $T_{m}$ can be expressed as:*

$$D_{n}\left(T_{m}\right)=d_{1}^{n}\left(T_{m}\right)\vee d_{2}^{n}\left(T_{m}\right),$$

*where* $d_{1}^{n}\left(T_{m}\right)$ *and* $d_{2}^{n}\left(T_{m}\right)$ *have different expressions, which are determined by the property of n.*

*If n is an odd number, and if the equivalent model of data stream 1 is used after the last change, then data stream 1 arrives sooner than data stream 2 before the first exchange. If the equivalent model of data stream 2 is used after the last change, then data stream 2 arrives sooner than data stream 1 before the first exchange. Therefore, we have:*

$$\begin{array}{cc} d_{1}^{n}\left(T_{m}\right)\leq & {Max}_{1\leq \theta_{2}\leq \cdots \leq \theta_{n}\leq m}\left[\varphi \left(m,\theta_{n},1\right)+\varphi \left(\theta_{n},\theta_{n-1},2\right)+\cdots +\varphi \left(\theta_{3},\theta_{2},1\right)+d_{2}^{0}\left(T_{\theta_{2}}\right)+T_{\theta_{2}}-T_{m}\right]\vee \\ & {Max}_{1\leq \theta_{1}\leq \theta_{2}\leq \cdots \leq \theta_{n}\leq m}\left[\varphi \left(m,\theta_{n},1\right)+\varphi \left(\theta_{n},\theta_{n-1},2\right)+\cdots +\varphi \left(\theta_{2},\theta_{1},2\right)+d_{1}^{0}\left(T_{\theta_{1}}\right)+T_{\theta_{1}}-T_{m}\right]\vee \\ & d_{2}^{0}\left(T_{m}\right)\vee d_{1}^{0}\left(T_{m}\right), \end{array}$$

*And:*

$$\begin{array}{cc} d_{2}^{n}\left(T_{m}\right) & \leq {Max}_{1\leq \theta_{2}\leq \cdots \leq \theta_{n}\leq m}\left[\varphi \left(m,\theta_{n},2\right)+\varphi \left(\theta_{n},\theta_{n-1},1\right)+\cdots +\varphi \left(\theta_{3},\theta_{2},2\right)+d_{1}^{0}\left(T_{\theta_{2}}\right)+T_{\theta_{2}}-T_{m}\right]\\ & \vee {Max}_{1\leq \theta_{1}\leq \theta_{2}\leq \cdots \leq \theta_{n}\leq m}\left[\varphi \left(m,\theta_{n},2\right)+\varphi \left(\theta_{n},\theta_{n-1},1\right)+\cdots +\varphi \left(\theta_{2},\theta_{1},1\right)+d_{2}^{0}\left(T_{\theta_{1}}\right)+T_{\theta_{1}}-T_{m}\right]\\ & \vee d_{2}^{0}\left(T_{m}\right)\vee d_{1}^{0}\left(T_{m}\right). \end{array}$$

*If n is an even number, and if the equivalent model of data stream 1 is used after the last change, then data stream 2 arrives sooner than data stream 1 before the first exchange. If the equivalent model of data stream 2 is used after the last change, then data stream 1 arrives sooner than data stream 2 before the first exchange. Therefore, we have:*

$$\begin{array}{c} d_{1}^{n}\left(T_{m}\right)\leq {Max}_{1\leq \theta_{2}\leq \cdots \leq \theta_{n}\leq m}[\varphi \left(m,\theta_{n},1\right)+\varphi \left(\theta_{n},\theta_{n-1},2\right)+\cdots +\varphi \left(\theta_{3},\theta_{2},1\right)+\\ d_{2}^{0}\left(T_{\theta_{2}}\right)+T_{\theta_{2}}-T_{m}]\vee {Max}_{1\leq \theta_{1}\leq \theta_{2}\leq \cdots \leq \theta_{n}\leq m}[\varphi \left(m,\theta_{n},1\right)+\varphi \left(\theta_{n},\theta_{n-1},2\right)+\cdots +\\ \varphi \left(\theta_{2},\theta_{1},2\right)+d_{1}^{0}\left(T_{\theta_{1}}\right)+T_{\theta_{1}}-T_{m}]\vee d_{2}^{0}\left(T_{m}\right)\vee d_{1}^{0}\left(T_{m}\right), \end{array}$$

*And:*

$$\begin{array}{c} d_{2}^{n}\left(T_{m}\right)\leq {Max}_{1\leq \theta_{2}\leq \cdots \leq \theta_{n}\leq m}[\varphi \left(m,\theta_{n},2\right)+\varphi \left(\theta_{n},\theta_{n-1},1\right)+\cdots +\varphi \left(\theta_{3},\theta_{2},2\right)+\\ d_{1}^{0}\left(T_{\theta_{2}}\right)+T_{\theta_{2}}-T_{m}]\vee {Max}_{1\leq \theta_{1}\leq \theta_{2}\leq \cdots \leq \theta_{n}\leq m}[\varphi \left(m,\theta_{n},2\right)+\varphi \left(\theta_{n},\theta_{n-1},1\right)+\cdots +\\ \varphi \left(\theta_{2},\theta_{1},1\right)+d_{2}^{0}\left(T_{\theta_{1}}\right)+T_{\theta_{1}}-T_{m}]\vee d_{2}^{0}\left(T_{m}\right)\vee d_{1}^{0}\left(T_{m}\right). \end{array}$$

The proof of Theorem 2 is given in the Appendix A (see, Appendix A.3).

In the above theorem, the system’s delay upper bound of the n-th exchange of data arrival sequence before time $T_{m}$ is obtained. Next, for any given time t, we drive the expression of system’s delay upper bound. 

Let us define $h\left(\alpha ,\beta ,t\right)=sup_{0\leq s\leq t}\left\{inf_{\tau \geq 0}\left\{\alpha \left(s\right)\leq \beta \left(s+\tau \right)\right\}\right\}$.

**Theorem** **3.** *At any given time t, the system’s delay upper bound can be expressed as d(t)≤h(α,β,t).*

The proof of Theorem 2 is given in Appendix A; see, Appendix A.4.

According to Theorem 3, we have $h\left(\alpha ,\beta ,t_{1}\right)\leq h\left(\alpha ,\beta ,t_{2}\right),\;t_{1}\leq t_{2}$. According to Theorem 3, $D_{n}\left(t\right)$ in Theorem 2 can be written as follows (n is an odd number):

$$\begin{array}{c} D_{n}\left(T_{m}\right)=d_{1}^{n}\left(T_{m}\right)\vee d_{2}^{n}\left(T_{m}\right)\leq h\left(\alpha_{1},\beta_{R1},T_{m}\right)\vee h\left(\alpha_{2},\beta_{R2},T_{m}\right)\vee \\ {Max}_{1\leq \theta_{1}\leq \theta_{2}\leq \cdots \leq \theta_{n}\leq m}[\varphi \left(m,T_{\theta_{n}},1\right)\varphi \left(T_{\theta_{n}},T_{\theta_{n-1}},1\right)+\cdots +\varphi \left(T_{\theta_{2}},T_{\theta_{1}},2\right)+\\ h\left(\alpha_{1},\beta_{R1},T_{\theta_{1}}\right)+T_{\theta_{1}}-T_{m}]\vee {Max}_{1\leq \theta_{1}\leq \theta_{2}\leq \cdots \leq \theta_{n}\leq m}[\varphi \left(m,\theta_{n},1\right)+\varphi \left(\theta_{n},\theta_{n-1},2\right)+\cdots +\\ \varphi \left(\theta_{2},\theta_{1},1\right)+h\left(\alpha_{2},\beta_{R2},T_{\theta_{1}}\right)+T_{\theta_{1}}-T_{m}]. \end{array}$$

### 3.4. The Method of Calculation of the Upper Bound Equivalent Synchronization System Delay

Furthermore, consider the case of p-channel data, if it needs to be synchronized. The flows are represented as 1,2, ⋯, p. According to the similar analysis in Section 3.2 and Section 3.3, the upper bound of the delays can be represented using the model as follows:

$$\begin{array}{c} D_{n}\left(T_{m}\right)=d_{1}^{n}\left(T_{m}\right)\vee d_{2}^{n}\left(T_{m}\right)\vee \cdots \vee d_{p}^{n}\left(T_{m}\right)\leq \\ {Max}_{1\leq \theta_{1}\leq \theta_{2}\leq \cdots \leq \theta_{n-1}\leq \theta_{n}\leq m,x_{k}\neq x_{k+1}\left(1\leq x_{k}\leq p\right)}[\varphi \left(m,T_{\theta_{n}},x_{n}\right)+\varphi \left(T_{\theta_{n}},T_{\theta_{n-1}},x_{n-1}\right)+\cdots +\\ \varphi \left(T_{\theta_{2}},T_{\theta_{1}},x_{2}\right)+h\left(\alpha_{x_{1}},\beta_{x_{1}},T_{\theta_{1}}\right)+T_{\theta_{1}}-\\ T_{m}]\vee h\left(\alpha_{1},\beta_{R1},T_{m}\right)\vee h\left(\alpha_{2},\beta_{R2},T_{m}\right)\vee \cdots \vee h\left(\alpha_{p},\beta_{Rp},T_{m}\right). \end{array}$$

We define:

$$\begin{array}{c} f\left(t\right)=\{1\leq \theta_{1}\leq \theta_{2}\leq \cdots \leq \theta_{n-1}\leq \theta_{n}\leq m,\;x_{k}\neq x_{k+1}\left(1\leq x_{k}\leq p\right)|\varphi \left(m,T_{\theta_{n}},x_{n}\right)+\\ \varphi \left(T_{\theta_{n}},T_{\theta_{n-1}},x_{n-1}\right)+\cdots +\varphi \left(T_{\theta_{2}},T_{\theta_{1}},x_{2}\right)+h\left(\alpha_{x_{1}},\beta_{Rx_{1}},T_{\theta_{1}}\right)+T_{\theta_{1}}\}. \end{array}$$

**Figure 2:**
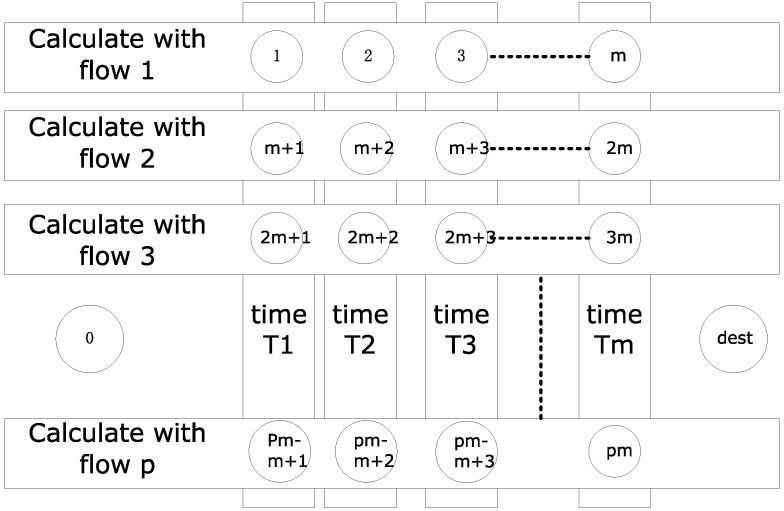
Finding the upper bound of delay.

Therefore:

$$D_{n}\left(T_{m}\right)\leq \left(Maxf\left(T_{m}\right)-T_{m}\right)\vee h\left(\alpha_{1},\beta_{R1},T_{m}\right)\vee h\left(\alpha_{2},\beta_{R2},T_{m}\right)\vee \cdots \vee h\left(\alpha_{p},\beta_{Rp},T_{m}\right),$$

where f(t) can be equivalent to any path from node 0 to node *dest* in Figure 2. Maxf(t) can be equivalent to obtaining the maximum path.

There are pm+2 nodes in this graph, and the nodes in the same row do not connect with each other. There is no connection between the nodes of the same column, but there is a connection between the two nodes in different rows and different columns. Besides, node 0 and any other node are connected. Node *dest* is connected to the node km only, and the distance is 0. The defined distance is represented as $l\left(node_{1},node_{2}\right)$. So, we have:

$$l\left(0,km+u\right)=h\left(\alpha_{x_{k}},\beta_{Rx_{k}},T_{u}\right)+T_{u},\;\;\forall 0\leq k\leq p-1,1\leq u\leq m,$$

l(km, dest)=0,  ∀1≤k≤p,

l(km+u,rm+v)=φ(v,u,r+1), ∀0≤r,k≤p−1, 1≤u<v≤m,

$$\varphi \left(v,u,r\right)=\{\begin{array}{cc} inf\left\{\tau \geq 0,\overset{v}{\underset{k=u+1}{\sum }}G_{r}\left(T_{k}\right)\leq \beta_{Rr}^{2}\left(\tau \right)\right\}, & \left(v\geq u+2\right),\\ 0, & else. \end{array}$$

In Figure 2, all paths from node 0 to node *dest* constitute the value of f(t). For example, taking $\theta_{1}=1$, $\theta_{2}=2$, $\theta_{3}=\theta_{4}=\cdots =\theta_{n}=\theta_{2}$, $x_{1}=1$, $x_{2}=3$, $x_{3}=m$, we have:

$$f\left(T_{m}\right)=h\left(\alpha_{1},\beta_{R1},T_{u}\right)+\varphi \left(2,1,3\right)+\varphi \left(m,2,p\right)+T_{1}-T_{m}.$$

It can be expressed as the path distance which is l(0,1)+l(1,2m+2)+l(2m+2,pm).

## 4. Monitoring System Delay Experimental Tests

### 4.1. Network Topology Simulation and Experimental Design

**Figure 3:**
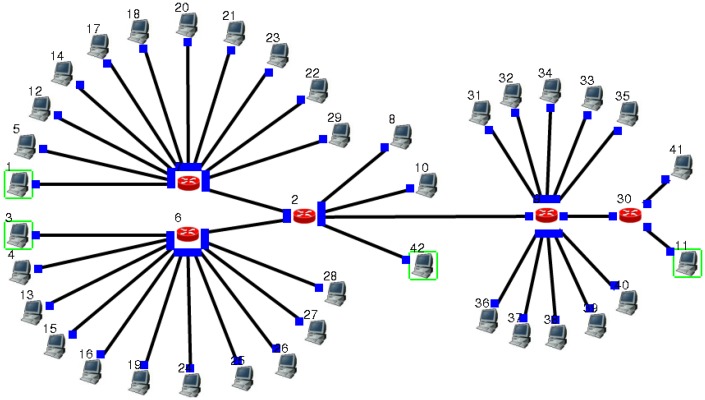
Network topology diagram of the synchronization delay test.

The network simulation software EstiNet is used for the experiments. For the sake of simplicity and generality, this paper selects the network topology structure shown in Figure 3. In order to simulate the time delay of the synchronization system, the data transmission introduced in Algorithm 1 is carried out in the network of Figure 3.


**Algorithm 1:**
1. Node 1 and 3 send packets in size of 100 kb at intervals of one second to node 42, denoting arrived curves as $\alpha_{1}\left(t\right)$ and $\alpha_{2}\left(t\right)$.2. Send node 42 packets of data to node 11 after synchronization.3. No. 5, 12, 14, 17, 18, 20, 21, 23, 22, 29 nodes send competing data packets to node 8, to constitute competing flow $R_{3}\left(t\right)$, which reaches curve $\alpha_{3}\left(t\right)$.4. No. 4, 13, 15, 16, 19, 24, 25, 26, 27, 28 nodes send competing data packets to node 8, to constitute competing flow $R_{4}\left(t\right)$, which reaches curve $\alpha_{4}\left(t\right)$.5. No. 31, 32, 33, 34, 35, 36, 37, 38, 39, 40 nodes send competing data packets to node 8, to constitute competing flow $R_{5}\left(t\right)$, which reaches curve $\alpha_{5}\left(t\right)$.6. All the links take bandwidth of 10 Mb. For all routers, the same configuration is used.

**Figure 4:**
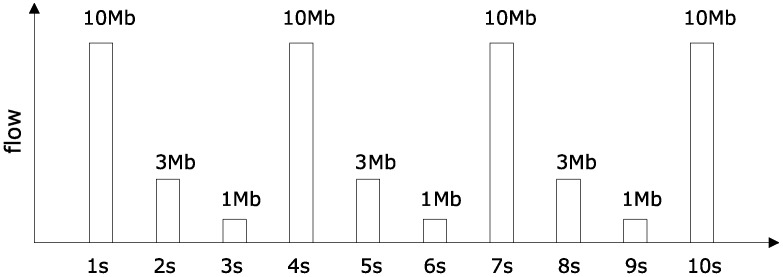
Superimposed competing flows $R_{3}\left(t\right)$.

In order to assess the feasibility and effectiveness of the proposed method, we design different $\alpha_{3}\left(t\right)$, $\alpha_{4}\left(t\right)$, $\alpha_{5}\left(t\right)$ in the experiment, which are given as follows. Deploying data generating program, such that data packets sent by $R_{3}$ are shown in Figure 4.

.

**Figure 5:**
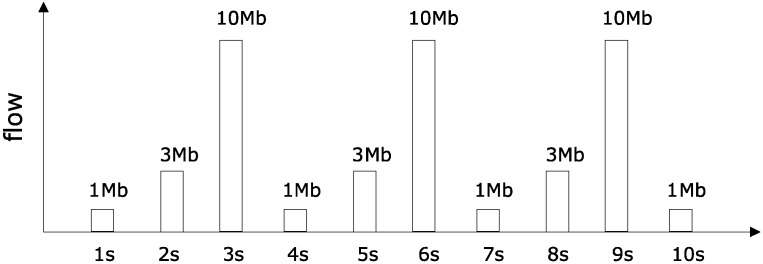
Superimposed competing flows $R_{4}\left(t\right)$.

Deploying data generating program, such that data packets sent by $R_{4}$ are shown in Figure 5.

**Figure 6:**
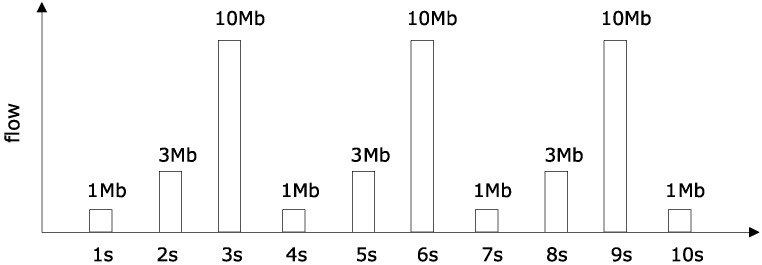
Superimposed competing flows $R_{5}\left(t\right)$.

Deploying data generating program, such that data packets sent by $R_{5}$ are shown in Figure 6.

Make competing flows $R_{3}\left(t\right)$, $R_{4}\left(t\right)$ and $R_{5}\left(t\right)$ consistent with data in BC-pAug89 [23] dataset. The *stg-trace file* command in software EstiNet can read network flows generated by the specified file. 

### 4.2. Theoretical Analysis of Delay Bound for Monitoring System

#### 4.2.1. First, the Computation Service Curve and the Scaling Function of Node 42 of the Synchronous Link Are Given

For simplicity, the synchronization process is to add the number of packets sent by node 1 and node 3. Then, the scaling function can be expressed as $S\left(a\right)=\frac{1}{2}a$. Further testing the processing time of a char type data. The total time of one million operations is less than 10 milliseconds, which means the average time for a single operation is less than 0.01 microseconds. In addition, defining the time complexity as O(n), thus the equivalent service curve can be expressed as C(t)≥100t×8=800t, where t is in units of microseconds, and C(t) is in units of byte.

#### 4.2.2. When There Are no Competing flows $\alpha_{3}\left(t\right)$, $\alpha_{4}\left(t\right)$ and $\alpha_{5}\left(t\right)$

Because the propagation delay of the link is set relatively small (1 microsecond, which is negligible), the delay of the system is mainly composed of processing delay, transfer delay and queuing delay. The equivalent service model using common links and routers is as follows:

$$\beta_{R,T}=R{\left[t-T\right]}^{+}=\{\begin{array}{ll} R\left(t-T\right), & t>T,\\ 0, & t\leq T. \end{array}$$

Since the propagation delay is small, assuming the router processing delay can be ignored, then the equivalent service model for each link and router is the bandwidth of the link. Therefore, the equivalent transport service curve from node 1 to node 42 is $\beta_{1,42}=\beta_{1,2}⊗\beta_{2,42}$, where $\beta_{1,2}=10t$. Based on the remaining service theorem [7], we have:

$$\beta_{2,42}=10t-\alpha_{2}\left(t\right),$$

$$\beta_{3,42}=10t-\alpha_{1}\left(t\right).$$

The equivalent transport service curve from node 11 to node 42 is $\beta_{42,11}=10t$. Due to the same amount of data in node 1 and node 3, and according to the previously obtained computing services curve C(t) of node 9 and scaling function S, we can obtain the equivalent service curve on node 1 and node 3 to node 11 as follows:

$$\beta_{3,11}=\beta_{1,11}=\beta_{1,2}⊗\beta_{2,42}⊗\frac{1}{2}\left(C⊗\underline{S^{-1}}\left(\beta_{42,11}\right)\right)=10t-\alpha_{1}\left(t\right).$$

#### 4.2.3. When Adding Competing Flows of $\alpha_{3}\left(t\right)$, $\alpha_{4}\left(t\right)$ and $\alpha_{5}\left(t\right)$

According to the residual service curve theorem [7], the service curves of node 1 and node 3 to node 2 are as follows:

$$\beta_{1,2}^{\prime }=10t-\alpha_{3}\left(t\right),$$

$$\beta_{3,2}^{\prime }=10t-\alpha_{4}\left(t\right).$$

The service curve from node 42 to node 11 is:

$$\beta_{42,11}^{\prime }=10t-\alpha_{5}\left(t\right).$$

Thus, the equivalent service curves on node 1 and node 3 to node 11 are as follows:

$$\beta_{1,11}^{\prime }=\beta_{1,2}^{\prime }⊗\beta_{2,42}⊗\frac{1}{2}\left(C⊗\underline{S^{-1}}\left(\beta_{42,11}^{\prime }\right)\right),$$

$$\beta_{3,11}^{\prime }=\beta_{3,42}^{\prime }⊗\beta_{3,42}⊗\frac{1}{2}\left(C⊗\underline{S^{-1}}\left(\beta_{42,11}^{\prime }\right)\right).$$

#### 4.2.4. Time Delay Increasing due to Forwarding

Because we adopt socket for packet forwarding in the implementation process, which means the packets will not be sent to node 11 until the total packets arrive to node 42. Nevertheless, each arrived packet should be forwarded in normal condition. Thus, the additional time delay is the transmission delay for the same size of the data packet from node 2 to node 11. Because the link bandwidth is 10 MB, the increase of delay can be obtained with a packet size/bandwidth. Define the delay as Δt. The system delay should be superimposed on Δt in the original calculation of boundary value. Namely, $D_{n}\left(t\right)=D_{n}\left(t\right)+\Delta t.$

Taking experimental verification of the above analysis, we get 22,625.8 microseconds of delay for 100 Kb packet to be sent from node 1 to node 11 transiting in node 42, and the delay directly sent from node 1 to node 11 is 12,896.8 μs, where Δt=9996.9≈100 Kb/10 Mbps.

Furthermore, multiple data links are considered to be forwarded. Therefore, the total delay is required to compose of the delay time for the last packet forwarded through an intermediate transmission. Intermediate delay δ can be calculated by:

δ=MTU size×(m−1)/10 Mbps.

#### 4.2.5. Arrival Flow Curve

(a) Arrival flow curve of monitoring sensors.

Since 100 kb monitoring data packet is sent, we can omit the time required for data transmission. The arrival flow curve on node 1 and node 3 can be represented by the following step function [14]:

$$\alpha_{1}\left(t\right)=⎡\frac{t}{T}⎤\times 100\;k,$$

$$\alpha_{2}\left(t\right)=⎡\frac{t}{T}⎤\times 100\;k.$$

where time t is in units of microseconds, and $T=10^{6}$.

(b) Competing arrival flow curve according to Figure 4, Figure 5 and Figure 6.

Maximum flow within any one second is 10 Mb for competition flow $R_{3}$ and $R_{4}$. Maximum flow within any two seconds is 13 Mb. Arbitrary maximum flow within three seconds is 14 Mb. So, we can get the curve as:

$$\alpha_{3}\left(t\right)=\alpha_{4}\left(t\right)=⎡\frac{t}{3T}⎤\times 10M+⎣\frac{t+2T}{3T}⎦\times 3M+⎣\frac{t+T}{3T}⎦\times 1M.$$

While for competing flow $R_{5},$ the largest flow within any one second is 1 Mb. Therefore, it can be expressed as:

$$\alpha_{5}\left(t\right)=⎡\frac{t}{T}⎤\times 1M.$$

Furthermore, since the network bandwidth is 10 Mbps, the data packet size of any transmission time segment x is $S_{x}\leq 10x$. We can further get constrained conditions for $\alpha_{3}^{\prime }\left(t\right),$ $\alpha_{4}^{\prime }\left(t\right),$ $\alpha_{5}^{\prime }\left(t\right)$ as follows:

$$\sigma =t/\left(3\times 10^{6}\right),$$

$$\tau =t\%\left(3\times 10^{6}\right),$$

ϑ(t)={σ×14M+10τ,(0≤τ≤106),σ×14M+10M+10(τ−10^6),(106<τ≤2×106),σ×14M+13M+10(τ−2×10^6),(2×106<τ≤3×106),

$$\alpha_{3}^{\prime }\left(t\right)=\alpha_{4}^{\prime }\left(t\right)=Min\left(\alpha_{3}\left(t\right),\vartheta \left(t\right)\right)=Min\left(\alpha_{4}\left(t\right),\vartheta \left(t\right)\right),$$

$$\alpha_{5}^{\prime }\left(t\right)=Min\left(\alpha_{5}\left(t\right),10t\right).$$

(c) Competing flow using BC-pAug89 data set.

We describe the competing flow using flow model under Gaussian assumption, and we have:

$$\alpha_{3}\left(t\right)=\alpha_{4}\left(t\right)=\alpha_{5}\left(t\right)=\rho n+k\sqrt{n\sigma^{2}+2\sigma^{2}\times \overset{n-1}{\underset{i=1}{\sum }}\left(n-i\right){\left(1+n^{\alpha }\right)}^{-\frac{\beta }{\alpha }}}.$$

If there is no other flow in the system, ∀t>0, we have:

$$h\left(\alpha_{1}\left(t\right),\beta_{1}\left(t\right),t\right)=h\left(\alpha_{2}\left(t\right),\beta_{2}\left(t\right),t\right)=\frac{200k}{10}=20,000,$$

$$\Delta t=\frac{100k}{10M}\times 10^{6}=10,000,$$

$$\delta =\frac{1500*8*6}{10M}\times 10^{6}\approx 6866,$$

$$\varphi \left(T_{\theta_{p}},T_{\theta_{q}},1\right)=\varphi \left(T_{\theta_{p}},T_{\theta_{q}},2\right)=\frac{100k\times \left(p-q\right)}{10}.$$

According to the equivalent calculating method in Section 3.4, we have $D_{n}\left(t\right)\leq h\left(\alpha_{1}\left(t\right),\beta_{1,11}^{\prime },t\right)+\Delta t+\delta =1,436,866$.

Using the theoretical analysis of BC-pAug89, we get competitive flow model under the generalized Cauchy hypothesis by *Matlab* calculation:

$$\alpha_{3}\left(t\right)=\alpha_{4}\left(t\right)=\alpha_{5}\left(t\right)<t.$$

The maximum transmission flow per second does not exceed 1 Mb. Therefore:

$$h\left(\alpha_{1}\left(t\right),\beta_{1,11}^{\prime },T\right)=h\left(\alpha_{1}\left(t\right),\beta_{1,11}^{\prime }\left(t\right),2T\right)=\cdots =h\left(\alpha_{1}\left(t\right),\beta_{1,11}^{\prime }\left(t\right),nT\right)<\frac{200k}{9}=22,222,$$

$$\varphi \left(T_{\theta_{p}},T_{\theta_{q}},1\right)=\varphi \left(T_{\theta_{p}},T_{\theta_{q}},2\right)=\frac{100k\times \left(p-q\right)}{9}.$$

According to the equivalent calculating method in Section 3.4, we can obtain:

$$D_{n}\left(t\right)\leq h\left(\alpha_{1}\left(t\right),\beta_{1,11}^{\prime },t\right)+\Delta t+\delta =22,222+10,000+6866=39,088.$$

### 4.3. Simulation Results of Monitoring System Delay

**Figure 7:**
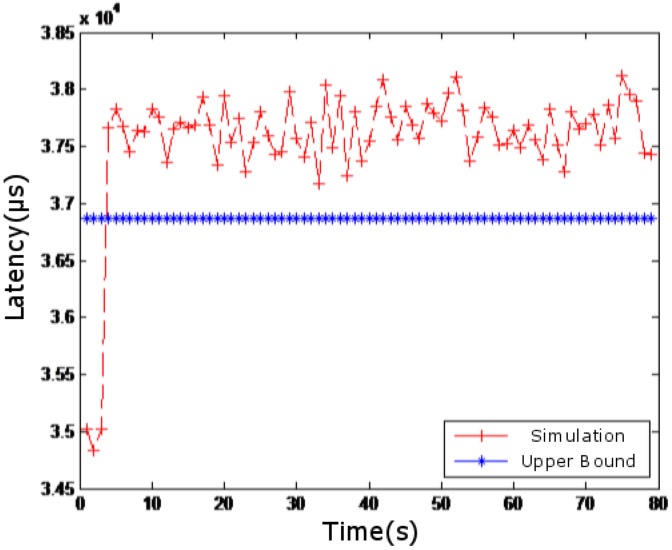
Results without competing flow.

**Figure 8:**
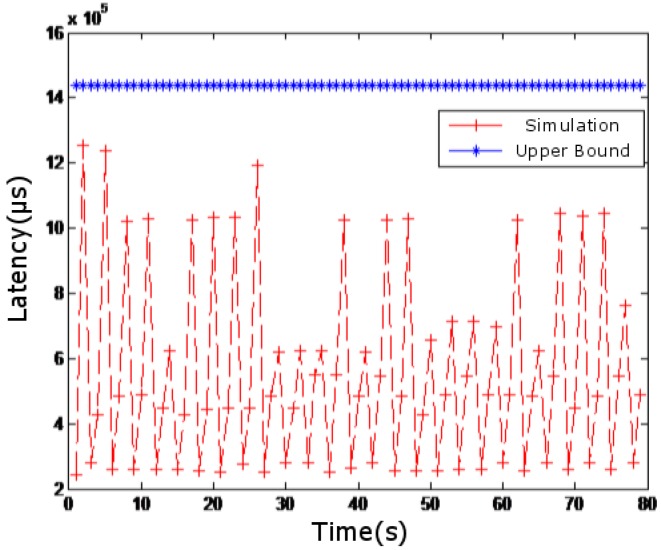
Results superimposing competing flows in Figure 4, Figure 5 and Figure 6.

**Figure 9:**
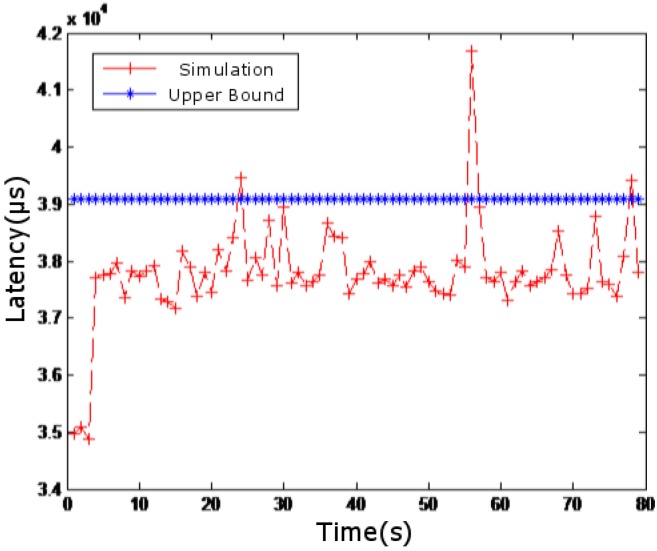
Results superimposing competing flows in BC-pAug89 data set.

In Figure 7, the simulated network delay is compared with the case where competing flow is not superimposed. In Figure 8, the simulated network delay is compared with the case where competing flows in Figure 4, Figure 5 and Figure 6 are superimposed. In Figure 9, the simulated network delay is compared with the case where competing flow in BC-pAug89 data set is superimposed.

Since delay caused by operations such as packet and packetization in the network communication and processing delay of the routers are not considered in the theoretical calculation, the upper bound of the theoretical delay calculated in Figure 7 and Figure 9 is smaller than the measured value. However, such deviation is within 5 milliseconds, which is quite small. According to Figure 7, Figure 8 and Figure 9, it can be seen that the theoretical results are close to those obtained by simulation, which indicates the feasibility and effectiveness of our proposed method.

## 5. Summary

In this paper, we propose a new method to provide an estimation for the upper bound of the smart grid monitoring system’s end-to-end delay. The graph theory approach is utilized to obtain the results, and simulations demonstrate the feasibility of the proposed method. It is notable that the main results in this paper are presented in forms of theorems. If the objectively existing system constraints and system parameter uncertainty are taken into consideration, the studied problem cannot be solved analytically. Instead, numerical methods, such as deep learning and reinforcement learning approaches, shall be applied to solve the new problem. On the other hand, with the development of smart grid technology, the concept of Energy Internet has developed rapidly in recent years [24,25]. Within the architecture of the Energy Internet, power systems and information systems are integrated, and energy and information are fused, such that a better scheduling and management of various kinds of energy can be achieved [3,25]. The latency problem of communication systems considered in this paper exists in the Energy Internet as well. In addition, the analysis of communication delay can be further extended to energy transmission systems [4,26]. In the future, we will conduct research on communication systems within scenarios of the Energy Internet.

## Appendix A.

### Appendix A.1. Proof of Theorem 1

**Proof.** We prove this theorem on two cases. (1) First, when $t>t_{2}$, assuming the new system’s backlog is $B^{\prime }\left(t\right)$ at time t, obviously, we have $B^{\prime }\left(t\right)\geq B\left(t\right)$. (a) Let us consider the case of $B^{\prime }\left(t\right)>B\left(t\right)$. For the suspension service system, the backlog for the system at time $t_{2}$ is $R\left(t_{2}\right)-R^{*}(t_{1})$. For the service system which is not suspended, the backlog at time $t_{2}$ is $R\left(t_{2}\right)-R^{*}(t_{2})$. Hence, the incremental backlog is $R^{*}(t_{2})-R^{*}(t_{1})$ at time $t_{2}$, and this term is defined as a suspensive backlog caused by the suspension. Under the same circumstances, we assume the process is after suspensive backlog. The server will only deal with the suspensive backlog in the idle moment. If the system suspensive backlog increases at time t, then we claim the suspensive backlog has not been finished yet, which means that the system is not idle from $t_{2}$ to t. Since $B^{\prime }\left(t\right)>B\left(t\right)\geq 0$, the system will still be busy until $B^{\prime }\left(t\right)$ has been finished processing. Afterwards, whether the system is busy or not is not taken into consideration. Assuming that $B^{\prime }\left(t\right)$ finishes at time t+τ, then $d^{\prime }\left(t\right)=\tau$. Since the system has always been busy from $t_{2}$ to t+τ, the amount of data that can be processed is at least $\beta \left(t+\tau -t_{2}\right)$, while the actual amount of data from $t_{2}$ to t+τ is $R\left(t\right)-R^{*}(t_{1})$. Then, we have:

$$\beta \left(t+\tau -t_{2}\right)\leq R\left(t\right)-R^{*}(t_{1}).$$

Since β is a generalized increment function, we have:

$$\tau \leq \left[inf\{\tau \geq 0,\;R\left(t\right)-R^{*}(t_{1})\leq \beta \left(\tau \right)\}+t_{2}-t\right].$$

 (b) Let us consider the case of $B^{\prime }\left(t\right)=B\left(t\right)$. System backlog at time t is not changed, which means the system delay at time t is unchanged, i.e., $d^{\prime }\left(t\right)=d\left(t\right).$ If we combine the above two situations, we obtain:

$$d^{\prime }\left(t\right)=d\left(t\right)\vee \tau \leq d\left(t\right)\vee \left[inf\{\tau \geq 0,\;R\left(t\right)-R^{*}(t_{1})\leq \beta \left(\tau \right)\}+t_{2}-t\right],\forall t>t_{2}.$$

(2) Second, we focus on the situation when $t_{1}<t<t_{2}$. Since the system was suspended from $t_{1}$ to $t_{2}$, the traffic flow within $t_{1}$ to $t_{2}$ will start to be processed after $t_{2}$. Therefore, the completion time $t_{fin}$ of its processing satisfies:

$$t_{fin}\leq inf\{\tau \geq 0,\;R\left(t\right)-R^{*}(t_{1})\leq \beta \left(\tau \right)\}+t_{2}.$$

So, we obtain the delay as:

$$d^{\prime }\left(t\right)=t_{fin}-t=\left[inf\{\tau \geq 0,\;R\left(t\right)-R^{*}(t_{1})\leq \beta \left(\tau \right)\}+t_{2}-t\right],$$

which finishes the proof.  □

### Appendix A.2. Discussions for the Value of m in $T_{m}$

(a) For the case $m=x_{1}+1$, we derive the upper bound of system delay $d_{1}^{1}\left(T_{m}\right).$

i If $t^{\prime }>T_{x_{1}}+d_{2}^{0}\left(T_{x_{1}}\right)$, data of $R_{1}$ with time scale $T_{m}$ arrives at the synchronization link after the data with the previous time scale is processed. It means that at time $t^{\prime }$, $\beta_{R1}^{2}$’s backlog would not increase. According to Theorem 1, $d_{1}^{1}\left(T_{m}\right)=d_{1}^{0}\left(T_{m}\right).$
ii If $t^{\prime }<T_{x_{1}}+d_{2}^{0}\left(T_{x_{1}}\right)$, according to the rules that first come first served, for $\beta_{R1}^{2}$, data will not be processed until time $T_{x_{1}}+d_{2}^{0}\left(T_{x_{1}}\right)$. Since data of $R_{2}$ arrives first, data of $R_{2}$ with time scale $T_{m}$ has arrived at time $t^{\prime }$. $\beta_{R2}^{2}$ would have to wait until $T_{x_{1}}+d_{2}^{0}\left(T_{x_{1}}\right)$ before processing the data. The system delay can be expressed by the equivalent delay of $R_{2}$, and we have $d_{1}^{1}\left(T_{m}\right)=d_{2}^{0}\left(T_{m}\right).$

According to Case i and Case ii, if $m=x_{1}+1$, the upper bound of system delay can be expressed as $d_{1}^{1}\left(T_{m}\right)=d_{1}^{0}\left(T_{m}\right)\vee d_{2}^{0}\left(T_{m}\right).$

(b) For the case $m\geq x_{1}+2$, we derive the upper bound of system delay $d_{1}^{1}\left(T_{m}\right).$

 iii If $t^{\prime }>T_{x_{1}}+d_{2}^{0}\left(T_{x_{1}}\right)$ at time $t^{\prime }$, the backlog of $\beta_{R1}^{2}$ may have increased. If so, according to Theorem 1, we can obtain the delay bound Δ as:

$$\Delta =inf\left\{\tau \geq 0,R_{1}^{1}\left(t^{\prime }\right)-R_{1}^{*}\left(T_{x_{1}}+d_{1}^{0}\left(T_{x_{1}}\right)\right)\leq \beta_{R1}^{2}\left(\tau \right)\right\}+T_{x_{1}}+d_{2}^{0}\left(T_{x_{1}}\right)-t^{\prime }.$$

Since $R_{1}^{1}\left(t^{\prime }\right)$ and $R_{1}\left(T_{m}\right)$ are identical, and they stand for the total delay of the data which were sent at time $T_{m}$ and passed by $\beta_{R1}^{2}$, we can obtain the data delay at time $T_{m}$ as:

$$d_{1}^{1}\left(T_{m}\right)=\vee +t^{\prime }-T_{m}.$$

Therefore, if $\beta_{R1}^{2}$’s backlog has increased at time $t^{\prime }$, then:

$$d_{1}^{1}\left(T_{m}\right)=\left[inf\left\{\tau \geq 0,R_{1}\left(T_{m}\right)-R_{1}^{*}\left(T_{x_{1}}+d_{1}^{0}\left(T_{x_{1}}\right)\right)\leq \beta_{R1}^{2}\left(\tau \right)\right\}+T_{x_{1}}+d_{2}^{0}\left(T_{x_{1}}\right)-T_{m}\right].$$

Conversely, if it hasn’t, then $d_{1}^{1}\left(T_{m}\right)=d_{1}^{0}\left(T_{m}\right)$. Since:

$$R\left(T_{m}\right)-R_{1}^{*}\left(T_{x_{1}}+d_{1}^{0}\left(T_{x_{1}}\right)\right)=\overset{m\left(m\geq x_{1}+2\right)}{\underset{k=x_{1}+1}{\sum }}G_{1}\left(T_{k}\right),$$

We have:

$$d_{1}^{1}\left(T_{m}\right)=d_{1}^{0}\left(T_{m}\right)\vee \left[inf\left\{\tau \geq 0,\overset{m\left(m\geq x_{1}+2\right)}{\underset{k=x_{1}+1}{\sum }}G_{1}\left(T_{k}\right)\leq \beta_{R1}^{2}\left(\tau \right)\right\}+T_{x_{1}}+d_{2}^{0}\left(T_{x_{1}}\right)-T_{m}\right].$$

 iv If $t^{\prime }<T_{x_{1}}+d_{2}^{0}\left(T_{x_{2}}\right)$, data will not be processed until $T_{x_{1}}+d_{2}^{0}\left(T_{x_{1}}\right)$, and the upper bound of the finishing time is:

$$t_{fin}=inf\left\{\tau \geq 0,R_{1}^{1}\left(t^{\prime }\right)-R_{1}^{*}\left(T_{x_{1}}+d_{1}^{0}\left(T_{x_{1}}\right)\right)\leq \beta_{R1}^{2}\left(\tau \right)\right\}+T_{x_{1}}+d_{2}^{0}\left(T_{x_{1}}\right).$$

Thereby, the system delay still satisfies:

$$d_{1}^{1}\left(T_{m}\right)=t_{fin}-T_{m},$$

$$d_{1}^{1}\left(T_{m}\right)=inf\left\{\tau \geq 0,\overset{m\left(m\geq x_{1}+2\right)}{\underset{k=x_{1}+1}{\sum }}G_{1}\left(T_{k}\right)\leq \beta_{R1}^{2}\left(\tau \right)\right\}+T_{x_{1}}+d_{2}^{0}\left(T_{x_{1}}\right)-T_{m}.$$

With the results in Case iii and Case iv, for the data sent at time $T_{m}$, its upper bound of end-to-end delay can be expressed as:

$$d_{1}^{1}\left(T_{m}\right)=d_{1}^{0}\left(T_{m}\right)\vee d_{2}^{0}\left(T_{m}\right)\vee \left[inf\left\{\tau \geq 0,\overset{m\left(m\geq x_{1}+2\right)}{\underset{k=x_{1}+1}{\sum }}G_{1}\left(T_{k}\right)\leq \beta_{R1}^{2}\left(\tau \right)\right\}+T_{x_{1}}+d_{2}^{0}\left(T_{x_{1}}\right)-T_{m}\right].$$

Apparently, for $\forall x>x_{1}$, $d_{2}^{0}\left(T_{x}\right)\leq d_{1}^{1}\left(T_{x}\right)$.

We define the following formulas:

$$\varphi \left(m,\theta ,1\right)=\{\begin{array}{cc} inf\left\{\tau \geq 0,\overset{m}{\underset{k=\theta +1}{\sum }}G_{1}\left(T_{k}\right)\leq \beta_{R1}^{2}\left(\tau \right)\right\}, & m\geq \theta +2,\\ 0, & else, \end{array}$$

$$\varphi \left(m,\theta ,2\right)=\{\begin{array}{cc} inf\left\{\tau \geq 0,\overset{m}{\underset{k=\theta +1}{\sum }}G_{2}\left(T_{k}\right)\leq \beta_{R2}^{2}\left(\tau \right)\right\}, & m\geq \theta +2,\\ 0, & else, \end{array}$$

$$\varphi \left(\theta_{m},\theta_{n},1\right)=\{\begin{array}{cc} inf\left\{\tau \geq 0,\overset{\theta_{n}}{\underset{k=\theta_{m}+1}{\sum }}G_{1}\left(T_{k}\right)\leq \beta_{R1}^{2}\left(\tau \right)\right\}, & \theta_{n}\geq \theta_{m}+2,\\ 0, & else, \end{array}$$

$$\varphi \left(\theta_{m},\theta_{n},2\right)=\{\begin{array}{cc} inf\left\{\tau \geq 0,\overset{\theta_{n}}{\underset{k=\theta_{m}+1}{\sum }}G_{2}\left(T_{k}\right)\leq \beta_{R2}^{2}\left(\tau \right)\right\}, & \theta_{n}\geq \theta_{m}+2,\\ 0, & else. \end{array}$$

Then, we have:

(A1) $$\varphi \left(\theta_{p},\theta_{m},2\right)+\varphi \left(\theta_{m},\theta_{n},1\right)=\{\begin{array}{ll} \varphi \left(\theta_{p},\theta_{n},1\right), & \left(\theta_{n}=\theta_{m}\right),\\ \varphi \left(\theta_{p},\theta_{m},2\right)+\varphi \left(\theta_{m},\theta_{n},1\right), & \left(\theta_{m}>\theta_{m}\right). \end{array}$$

From Case (a) and Case (b), for data with time scale $T_{m}$, its upper bound of delay can be expressed as:

$$d_{1}^{1}\left(T_{m}\right)=d_{1}^{0}\left(T_{m}\right)\vee d_{2}^{0}\left(T_{m}\right)\vee \left[\varphi \left(m,T_{x_{1}},1\right)+T_{x_{1}}+d_{2}^{0}\left(T_{x_{1}}\right)-T_{m}\right].$$

### Appendix A.3. Proof of Theorem 2

**Proof.** Using the mathematical induction, according to the analysis in Section 3.2, we have:

$$d_{1}^{1}\left(T_{m}\right)\leq {Max}_{1\leq \theta \leq m}\left[\varphi \left(m,\theta ,1\right)+d_{2}^{0}\left(T_{\theta }\right)+T_{\theta }-T_{m}\right]\vee d_{1}^{0}\left(T_{m}\right)\vee d_{2}^{0}\left(T_{m}\right),$$

$$d_{2}^{1}\left(T_{m}\right)\leq {Max}_{1\leq \theta \leq m}\left[\varphi \left(m,\theta ,2\right)+d_{1}^{0}\left(T_{\theta }\right)+T_{\theta }-T_{m}\right]\vee d_{1}^{0}\left(t\right)\vee d_{2}^{0}\left(T_{m}\right),$$

$$\begin{array}{cc} d_{2}^{2}\left(T_{m}\right)\leq & Max_{1\leq \theta_{2}\leq m}\left[\varphi \left(m,T_{\theta_{2}},2\right)+d_{1}^{0}\left(T_{\theta_{2}}\right)+T_{\theta_{2}}-T_{m}\right]\vee \\ & Max_{1\leq \theta_{1}\leq \theta_{2}\leq m}\left[\varphi \left(m,T_{\theta_{2}},2\right)+\varphi \left(T_{\theta_{2}},T_{\theta_{1}},1\right)+d_{2}^{0}\left(T_{\theta_{1}}\right)+T_{\theta_{1}}-T_{m}\right]\vee \\ & d_{1}^{0}\left(T_{m}\right)\vee d_{2}^{0}\left(T_{m}\right), \end{array}$$

$$\begin{array}{cc} d_{1}^{2}\left(T_{m}\right)\leq & Max_{1\leq \theta_{2}\leq m}\left[\varphi \left(m,T_{\theta_{2}},1\right)+d_{2}^{0}\left(T_{\theta_{2}}\right)+T_{\theta_{2}}-T_{m}\right]\vee \\ & Max_{1\leq \theta_{1}\leq \theta_{2}\leq m}\left[\varphi \left(m,T_{\theta_{2}},1\right)+\varphi \left(T_{\theta_{2}},T_{\theta_{1}},2\right)+d_{1}^{0}\left(T_{\theta_{1}}\right)+T_{\theta_{1}}-T_{m}\right]\vee \\ & d_{1}^{0}\left(T_{m}\right)\vee d_{2}^{0}\left(T_{m}\right). \end{array}$$

Assuming n is an odd number, and the following inequality is satisfied:

$$\begin{array}{c} d_{1}^{n-1}\left(T_{\omega }\right)\leq Max_{1\leq \theta_{2}\leq \cdots \leq \theta_{n-1}\leq \omega }[\varphi \left(\omega ,\theta_{n-1},1\right)+\cdots +\varphi \left(\theta_{3},\theta_{2},1\right)+d_{2}^{0}\left(T_{\theta_{2}}\right)+T_{\theta_{2}}-\\ T_{\omega }]\vee Max_{1\leq \theta_{1}\leq \theta_{2}\leq \cdots \leq \theta_{n-1}\leq \omega }\left[\varphi \left(\omega ,\theta_{n-1},1\right)+\cdots +\varphi \left(\theta_{2},\theta_{1},2\right)+d_{1}^{0}\left(T_{\theta_{1}}\right)+T_{\theta_{1}}-T_{\omega }\right]\vee \\ d_{1}^{0}\left(T_{\omega }\right)\vee d_{2}^{0}\left(T_{\omega }\right) \end{array}$$

$$\begin{array}{c} d_{2}^{n-1}\left(T_{\omega }\right)\leq Max_{1\leq \theta_{2}\leq \cdots \leq \theta_{n-1}\leq \omega }[\varphi \left(\omega ,\theta_{n-1},2\right)+\cdots +\varphi \left(\theta_{3},\theta_{2},2\right)+d_{1}^{0}\left(T_{\theta_{2}}\right)+T_{\theta_{2}}-\\ T_{\omega }]\vee Max_{1\leq \theta_{1}\leq \theta_{2}\leq \cdots \leq \theta_{n-1}\leq \omega }\left[\varphi \left(\omega ,\theta_{n-1},2\right)+\cdots +\varphi \left(\theta_{2},\theta_{1},1\right)+d_{2}^{0}\left(T_{\theta_{1}}\right)+T_{\theta_{1}}-T_{\omega }\right]\vee \\ d_{1}^{0}\left(T_{\omega }\right)\vee d_{2}^{0}\left(T_{\omega }\right). \end{array}$$

Consider exchanges for n times. First let us consider $d_{2}^{n}\left(t\right)$. It can be seen as equivalent to the output of $R_{2}$ through service $\beta_{R2}^{1}$ after the suspension of $\beta_{R2}^{2}$, where the pause period is $T_{x_{n}}+d_{2}^{0}\left(T_{x_{n}}\right)<t<T_{x_{2}}+d_{1}^{n-1}\left(T_{x_{n}}\right).$ We have:

$$\begin{array}{cc} d_{2}^{n}\left(T_{m}\right)\leq d_{2}^{0}\left(T_{m}\right)\vee & d_{1}^{n-1}\left(T_{m}\right)\vee {Max}_{1\leq \theta_{1}\leq \theta_{2}\leq \cdots \leq \theta_{n-1}\leq \theta_{n}\leq m}[\varphi \left(m,T_{\theta_{n}},2\right)+T_{\theta_{n}}\\ & +d_{1}^{n-1}\left(T_{\theta_{n}}\right)-T_{m}]. \end{array}$$

If we assume $d_{1}^{n-1}\left(T_{\theta_{n}}\right)=d_{2}^{0}\left(T_{\theta_{n}}\right)$, then $d_{2}^{n}\left(T_{m}\right)=d_{2}^{0}\left(T_{m}\right)$, and:

$$\begin{array}{c} Max_{1\leq \theta_{1}\leq \theta_{2}\leq \cdots \leq \theta_{n-1}\leq \theta_{n}\leq m}\left[\varphi \left(m,T_{\theta_{n}},2\right)+T_{\theta_{n}}+d_{1}^{n-1}\left(T_{\theta_{n}}\right)-T_{m}\right]\leq d_{2}^{0}\left(T_{m}\right)\vee \\ Max_{1\leq \theta_{n}\leq m}\left[\varphi \left(m,T_{\theta_{n}},2\right)+T_{\theta_{n}}+d_{1}^{0}\left(T_{\theta_{n}}\right)-T_{m}\right]\vee Max_{1\leq \theta_{2}\leq \cdots \leq \theta_{n-1}\leq \theta_{n}\leq m}[\varphi \left(m,T_{\theta_{n}},2\right)+\\ T_{\theta_{n}}+\varphi \left(T_{\theta_{n}},T_{\theta_{n-1}},1\right)+\cdots +\varphi \left(T_{\theta_{3}},T_{\theta_{2}},1\right)+d_{2}^{0}\left(T_{\theta_{2}}\right)+T_{\theta_{2}}-T_{\theta_{n}}-T_{m}]\vee \\ Max_{1\leq \theta_{1}\leq \theta_{2}\leq \cdots \leq \theta_{n-1}\leq \theta_{n}\leq m}[\varphi \left(m,T_{\theta_{n}},2\right)+T_{\theta_{n}}+\varphi \left(T_{\theta_{n}},T_{\theta_{n-1}},1\right)+\cdots +\varphi \left(T_{\theta_{2}},T_{\theta_{1}},2\right)+\\ d_{1}^{0}\left(T_{\theta_{1}}\right)+T_{\theta_{1}}-T_{\theta_{n}}-T_{m}]=d_{2}^{0}\left(T_{m}\right)\vee Max_{1\leq \theta_{1}\leq \theta_{2}\leq \cdots \leq \theta_{n-1}\leq \theta_{n}\leq m}[\varphi \left(m,T_{\theta_{n}},2\right)+T_{\theta_{n}}+\\ d_{1}^{0}\left(T_{\theta_{n}}\right)-T_{m}]\vee Max_{1\leq \theta_{2}\leq \cdots \leq \theta_{n-1}\leq \theta_{n}\leq m}[\varphi \left(m,T_{\theta_{n}},2\right)+\varphi \left(T_{\theta_{n}},T_{\theta_{n-1}},1\right)+\cdots +\\ \varphi \left(T_{\theta_{3}},T_{\theta_{2}},1\right)+d_{2}^{0}\left(T_{\theta_{2}}\right)+T_{\theta_{2}}-T_{m}]\vee Max_{1\leq \theta_{1}\leq \theta_{2}\leq \cdots \leq \theta_{n-1}\leq \theta_{n}\leq m}[\varphi \left(m,T_{\theta_{n}},2\right)+\\ \varphi \left(T_{\theta_{n}},T_{\theta_{n-1}},1\right)+\cdots +\varphi \left(T_{\theta_{2}},T_{\theta_{1}},2\right)+d_{1}^{0}\left(T_{\theta_{1}}\right)+T_{\theta_{1}}-T_{m}] \end{array}$$

Apparently, if $\theta_{1}=\theta_{2}=\cdots =\theta_{n-1}=\theta_{n}$, then:

$$\begin{array}{c} {Max}_{1\leq \theta_{1}=\theta_{2}=\cdots =\theta_{n-1}=\theta_{n}\leq m}[\varphi \left(m,T_{\theta_{n}},2\right)+\varphi \left(T_{\theta_{n}},T_{\theta_{n-1}},1\right)+\cdots +\varphi \left(T_{\theta_{3}},T_{\theta_{2}},1\right)+\\ d_{2}^{0}\left(T_{\theta_{1}}\right)+T_{\theta_{1}}-T_{m}]={Max}_{1\leq \theta_{n}\leq m}\left[\varphi \left(m,T_{\theta_{n}},2\right)+T_{\theta_{n}}+d_{1}^{0}\left(T_{\theta_{n}}\right)-T_{m}\right]. \end{array}$$

Therefore, we have:

$$\begin{array}{c} d_{2}^{n}\left(T_{m}\right)\leq d_{2}^{0}\left(t\right)\vee d_{1}^{n-1}\left(T_{m}\right)\vee Max_{1\leq \theta_{2}\leq \cdots \leq \theta_{n-1}\leq \theta_{n}\leq m}[\varphi \left(m,T_{\theta_{n}},2\right)+\varphi \left(T_{\theta_{n}},T_{\theta_{n-1}},1\right)++\\ \cdots +\varphi (T_{\theta_{3}},T_{\theta_{2}},1)d_{2}^{0}\left(T_{\theta_{2}}\right)+T_{\theta_{2}}-T_{m}]\vee Max_{1\leq \theta_{1}\leq \theta_{2}\leq \cdots \leq \theta_{n-1}\leq \theta_{n}\leq m}[\varphi \left(m,T_{\theta_{n}},2\right)+\\ \varphi :\left(T_{\theta_{n}},T_{\theta_{n-1}},1\right)+\cdots +\varphi \left(T_{\theta_{2}},T_{\theta_{1}},2\right)+d_{1}^{0}\left(T_{\theta_{1}}\right)+T_{\theta_{1}}-T_{m}]. \end{array}$$

Meanwhile, apparently:

$$\begin{array}{c} Max_{1\leq \theta_{2}\leq \cdots \leq \theta_{n-1}\leq \theta_{n}\leq m}[\varphi \left(m,T_{\theta_{n}},2\right)+\varphi \left(T_{\theta_{n}},T_{\theta_{n-1}},1\right)+\cdots +\varphi \left(T_{\theta_{3}},T_{\theta_{2}},1\right)+d_{2}^{0}\left(T_{\theta_{2}}\right)+\\ T_{\theta_{2}}-T_{m}]=Max_{1\leq \theta_{1}\leq \cdots \leq \theta_{n-1}\leq m}\left[\varphi \left(m,\theta_{n-1},1\right)+\cdots +\varphi \left(\theta_{3},\theta_{2},1\right)+d_{2}^{0}\left(T_{\theta_{2}}\right)+T_{\theta_{2}}-T_{m}\right]. \end{array}$$

If we set $\theta_{n-1}=\theta_{n}$, then:

$$\begin{array}{c} Max_{1\leq \theta_{1}\leq \theta_{2}\leq \cdots \leq \theta_{n-1}=\theta_{n}\leq m}[\varphi \left(m,T_{\theta_{n}},2\right)+\varphi \left(T_{\theta_{n}},T_{\theta_{n-1}},1\right)+\cdots +\varphi \left(T_{\theta_{2}},T_{\theta_{1}},2\right)+\\ d_{1}^{0}\left(T_{\theta_{1}}\right)+T_{\theta_{1}}-T_{m}]=Max_{1\leq \theta_{1}\leq \theta_{2}\leq \cdots \leq \theta_{n-1}\leq m}[\varphi \left(m,\theta_{n-1},1\right)+\cdots +\varphi \left(\theta_{2},\theta_{1},2\right)+\\ d_{1}^{0}\left(T_{\theta_{1}}\right)+T_{\theta_{1}}-T_{m}]. \end{array}$$

Thus, we have:

$$\begin{array}{c} d_{2}^{n}\left(T_{m}\right)\leq Max_{1\leq \theta_{2}\leq \cdots \leq \theta_{n-1}\leq \theta_{n}\leq m}[\varphi \left(m,T_{\theta_{n}},2\right)+\varphi \left(T_{\theta_{n}},T_{\theta_{n-1}},1\right)+\cdots +\varphi \left(T_{\theta_{3}},T_{\theta_{2}},1\right)+\\ d_{2}^{0}\left(T_{\theta_{2}}\right)+T_{\theta_{2}}-T_{m}]\vee Max_{1\leq \theta_{1}\leq \theta_{2}\leq \cdots \leq \theta_{n-1}\leq \theta_{n}\leq m}[\varphi \left(m,T_{\theta_{n}},2\right)+\varphi \left(T_{\theta_{n}},T_{\theta_{n-1}},1\right)+\cdots +\\ \varphi \left(T_{\theta_{2}},T_{\theta_{1}},2\right)+d_{1}^{0}\left(T_{\theta_{1}}\right)+T_{\theta_{1}}-T_{m}]\vee d_{2}^{0}\left(T_{m}\right)\vee d_{1}^{0}\left(T_{m}\right). \end{array}$$

In the same way, we can prove:

$$\begin{array}{c} d_{1}^{n}\left(T_{m}\right)\leq Max_{1\leq \theta_{2}\leq \cdots \leq \theta_{n}\leq m}[\varphi \left(m,\theta_{n},1\right)+\varphi \left(\theta_{n},\theta_{n-1},2\right)+\cdots +\varphi \left(\theta_{3},\theta_{2},2\right)+\\ d_{1}^{0}\left(T_{\theta_{2}}\right)+T_{\theta_{2}}-T_{m}]\vee Max_{1\leq \theta_{1}\leq \theta_{2}\leq \cdots \leq \theta_{n}\leq m}[\varphi \left(m,\theta_{n},1\right)+\varphi \left(\theta_{n},\theta_{n-1},2\right)+\cdots +\\ \varphi \left(\theta_{2},\theta_{1},1\right)+d_{2}^{0}\left(T_{\theta_{1}}\right)+T_{\theta_{1}}-T_{m}]\vee d_{2}^{0}\left(T_{m}\right)\vee d_{1}^{0}\left(T_{m}\right). \end{array}$$

When n is an even number, a similar proof can be obtained. Here, we omit the details.  □

### Appendix A.4. Proof of Theorem 3

**Proof.** The analytical methods are consistent with the proof of processing delays in the network calculus theory. For a given time t, taking τ≤d(t), we have $R\left(t\right)\geq R^{*}\left(t+\tau \right)$. Meanwhile, according to the definition of the service curve, we have:

$$R^{*}\left(t+\tau \right)\geq R\left(t+\tau \right)\vee \beta \left(t\right)=inf_{0\leq s\leq t+\tau }\left\{R\left(t+\tau -s\right)+\beta \left(s\right)\right\}.$$

Therefore, for $0\leq s_{0}\leq t+\tau$:

$$R^{*}\left(t+\tau \right)=R\left(t+\tau -s_{0}\right)+\beta \left(s_{0}\right).$$

Thus, we obtain:

$$R\left(t\right)\geq R\left(t+\tau -s_{0}\right)+\beta \left(s_{0}\right).$$

Furthermore, for $t+\tau -s_{0}\leq t$, we have:

$$R\left(t\right)-R\left(t+\tau -s_{0}\right)\leq \alpha \left(s_{0}-\tau \right).$$

Therefore:

$$\beta \left(s_{0}\right)\leq R\left(t\right)-R\left(t+\tau -s_{0}\right)\leq \alpha \left(s_{0}-\tau \right).$$

Define $inf_{\tau \geq 0}\left\{\alpha \left(s\right)\leq \beta \left(s+\tau \right)\right\}=\delta \left(s\right)$. We have $\tau \leq \delta \left(s_{0}-\tau \right)$. According to $0\leq s_{0}\leq t+\tau$, we get $s_{0}-\tau \leq t$. While:

$$h\left(\alpha ,\beta ,t\right)=sup_{0\leq s\leq t}\left\{inf_{\tau \geq 0}\left\{\alpha \left(s\right)\leq \beta \left(s+\tau \right)\right\}\right\},$$

We have τ≤h(α,β,t). Since it is true for any τ≤d(t), we have d(t)≤h(α,β,t).  □
